# Psychological impact and acceptability of magnetic resonance imaging and X-ray mammography: the MARIBS Study

**DOI:** 10.1038/bjc.2011.1

**Published:** 2011-02-15

**Authors:** J Hutton, L G Walker, F J Gilbert, D G Evans, R Eeles, G E Kwan-Lim, D Thompson, L J Pointon, D M Sharp, M O Leach

**Affiliations:** 1 North Lanarkshire Council, 73-77 Merry Street, Motherwell, Lanarkshire ML1 1JE, UK; 2 Institute of Rehabilitation, Postgraduate Medical Institute of the University of Hull and Hull York Medical School, 215 Anlaby Road, Kingston upon Hull HU3 2PG, UK; 3 Aberdeen Biomedical Imaging Centre, University of Aberdeen, Foresterhill, Aberdeen AB25 2ZD, UK; 4 Genetic Medicine, Manchester Academic Health Sciences Centre, Central Manchester University Hospitals NHS Foundation Trust, Manchester M13 9WL, UK; 5 The Institute of Cancer Research and Royal Marsden NHS Foundation Trust, Downs Road, Sutton, Surrey SM2 5PT, UK; 6 Cancer Research UK Clinical Magnetic Resonance Research Group, Institute of Cancer Research and Royal Marsden NHS Foundation Trust, Downs Road, Sutton, Surrey SM2 5PT, UK; 7 Cancer Research UK Genetic Epidemiology Unit, Department of Public Health & Primary Care, Strangeways Research Laboratory, University of Cambridge, Worts Causeway, Cambridge CB1 8RN, UK; 8 Early Drug Development Center, Dana Farber Cancer Institute, 44 Binney Street, Boston, MA 02115, USA

**Keywords:** MARIBS, psychology, MRI, anxiety, breast cancer

## Abstract

**Background:**

As part of the Magnetic Resonance Imaging for Breast Screening (MARIBS), Study women with a family history of breast cancer were assessed psychologically to determine the relative psychological impact and acceptability of annual screening using magnetic resonance imaging (MRI) and conventional X-ray mammography (XRM).

**Methods:**

Women were assessed psychologically at baseline (4 weeks before MRI and XRM), immediately before, and immediately after, both MRI and XRM, and at follow-up (6 weeks after the scans).

**Results:**

Overall, both procedures were found to be acceptable with high levels of satisfaction (MRI, 96.3% and XRM, 97.7% NS) and low levels of psychological morbidity throughout, particularly at 6-week follow-up. Low levels of self-reported distress were reported for both procedures (MRI, 13.5% and XRM, 7.8%), although MRI was more distressing (*P*=0.005). Similarly, higher anticipatory anxiety was reported before MRI than before XRM (*P*=0.003). Relative to XRM, MRI-related distress was more likely to persist at 6 weeks after the scans in the form of intrusive MRI-related thoughts (*P*=0.006) and total MRI-related distress (*P*=0.014). More women stated that they intended to return for XRM (96.3%) than for MRI (88% *P*<0.0005). These effects were most marked for the first year of screening, although they were also statistically significant in subsequent years.

**Conclusion:**

Given the proven benefits of MRI in screening for breast cancer in this population, these data point to the urgent need to provide timely information and support to women undergoing MRI.

Women with a known family history of breast cancer, or of a predisposing gene mutation such as *BRCA1* or *BRCA2*, have a cumulative lifetime risk of 45–65% (Antoniou *et al*, 2003), and a significant number of these cancers are diagnosed before the age of 50 years. A number of prevention strategies have been identified for these women, including bilateral prophylactic mastectomy, chemoprevention, and screening with annual X-ray mammography (XRM). Prophylactic mastectomy remains unacceptable to the majority of mutation carriers for a variety of reasons, including the prospect of mutilating surgery, loss of sensation, and breast cancer not being totally preventable (Warner, 2004). There is also some concern about the poor sensitivity of XRM because of the difficulties in evaluating X-ray mammograms from dense tissue in younger, premenopausal women and the concern that the tumours resulting from gene mutations may be particularly aggressive (Liberman, 2004).

There has been a growing interest in the use of breast magnetic resonance imaging (MRI), and a number of studies have been undertaken to assess the benefits of adding MRI to XRM in screening young women at high risk of breast cancer. A recent systematic review of these studies (Lord *et al*, 2007) found consistent evidence that adding MRI to XRM provides a highly sensitive screening strategy (sensitivity range: 93–100%) compared with XRM alone (25–59%) or with XRM plus ultrasound with or without clinical breast examination (25–59%). A cost-effectiveness analysis of the UK Magnetic Resonance Imaging for Breast Screening (MARIBS) study evaluated the incremental cost per cancer detected by XRM and XRM plus MRI, and concluded that adding MRI to XRM is potentially cost-effective for women at high familial risk of breast cancer, particularly for *BRCA1* and *BRCA2* mutation carriers (Griebsch *et al*, 2006). The compelling evidence for the benefits of MRI has translated into UK National Institute for Clinical Excellence (NICE) guidelines, which now recommend that women who are known mutation carriers, or who are at substantially increased familial risk, should be offered annual MRI surveillance (McIntosh *et al*, 2006).

A systematic review of the psychological impact of XRM for screening women with a family history of breast cancer concluded that women do not seem to experience high levels of anxiety associated with XRM, but may experience slight increases in the short term. Interpretation of studies evaluating the psychological impact of XRM, however, is hampered by the heterogeneity of the measures used, the use of non-validated measures, small sample sizes, and the lack of a baseline measure of anxiety uncontaminated by the knowledge of an impending screening appointment (Watson *et al*, 2005).

Two studies have compared the psychological effects of MRI plus XRM with XRM alone for screening women at high risk of breast cancer. In the Dutch study (Van Dooren *et al*, 2005), psychological distress remained within normal limits throughout screening for the group as a whole (*n*=357). However, elevated breast cancer-specific distress related to screening was found in women who did ‘excessive’ breast self-examination (defined as at least once per week), risk over-estimators, and women closely involved in the breast cancer care of a sister. In a sample of women from the Toronto study followed up over the course of 2 years, there was no evidence of any effect on global anxiety, depression, or breast cancer-related anxiety. However, only 57 women were recruited and, of those, 28 completed at least one assessment and 25 completed at least three (Warner, 2004).

Potentially, annual breast MRI could be extremely distressing. The procedure can take upwards of 50 min; the MRI scanner can be claustrophobic and noisy, and the positioning of the participant at an angle, higher in the bore, where the bore is narrower may make the procedure more claustrophobic (Anderson and Walker, 2002). Psychological reactions to MRI vary considerably and it has been reported that 25–37% of MRI participants experience moderate to severe claustrophobia (Katz *et al*, 1994; McIsaac *et al*, 1998).

The present study represents the largest and most comprehensive study to date of the psychological impact and acceptability of annual MRI plus XRM, compared with XRM alone, in women with a family history of breast cancer.

## Materials and methods

The MARIBS study, funded by the United Kingdom Medical Research Council, was a multicentre study to compare the sensitivity, specificity, cost-effectiveness, and psychosocial impact of annual XRM with XRM plus MRI in young women with a family history of breast cancer. The rationale, protocol, and outcome for the overall study have been reported, and the radiological and economic aspects have been detailed elsewhere (Brown *et al*, 2000a, 2000b; Leach *et al*, 2005; Warren *et al*, 2005, 2006a, 2006b; Griebsch *et al*, 2006; Gilbert *et al*, 2009)

Women between the age of 35 and 50 years were recruited between 1997 and 2004, from 22 Genetics Departments across the United Kingdom. To be eligible, women had to meet at least one of the following criteria: they had to be tested carriers of a deleterious *BRCA1*, *BRCA2*, or *TP53* mutation; be first-degree relative of someone with a *BRCA1*, *BRCA2*, or *TP53* mutation, or a strong family history of breast and/or ovarian cancer. All participants were offered annual screening with both contrast-enhanced MRI and XRM. X-ray mammography was performed according to the standards of the National Health Service Breast Screening Programme. Magnetic resonance imaging was undertaken using a specified protocol (Warren *et al*, 2005) and gadopentetate dimeglumine (Magnevist, Schering Healthcare, Burgess Hill, West Sussex, UK). Both XRM and MRI were independently double reported.

To determine the relative psychological impact and acceptability of MRI and XRM, women were assessed at baseline (by 4 weeks before MRI and XRM), immediately before both MRI and XRM, immediately after both MRI and XRM, and at follow-up (6 weeks after the scans). Booklets for each time point were professionally printed and contained standardised questionnaires to evaluate various psychosocial outcomes and *ad hoc* questions to document sociodemographic characteristics, previous experiences of mammography, intention to return for further screening, and patient satisfaction. (Copies of the booklets are available on application to the first author.) The booklets were printed in a format that permitted optical character recognition and automatic scoring. (The booklets were printed and scored by KendataPrint Services, Nutsay Lane, Totton, Southampton SO40 3NB, UK.)

The standardised questionnaires were as follows:

*The Hospital Anxiety and Depression Scale (HADS)* (Zigmond and Snaith, 1983): The HADS is a widely used, 14-item, self-report, screening tool for anxiety and depression. It is easy to administer and is well accepted, and has been designed to be especially relevant for the somatic medicine setting rather than mental health (Hermann, 1997). Its performance and psychometric properties have been established as a screening tool in a cancer setting (Walker *et al*, 2007), and it has been used previously to evaluate the psychosocial effects of breast screening in women with a family history of breast cancer within the context of the UK Breast Screening Programme (Gilbert *et al*, 1998). It is customary to analyse HADS data in two ways: mean scores (for the anxiety and depression scales) and total scores, and proportions of patients scoring in the ‘normal’ (0–7), ‘borderline’ (8–10), and ‘clinically significant’ (>11) ranges. Scores on each scale can range from 0 to 14.

*The Health Questionnaire (HQ)* (Walker *et al*, 1994): This is a seven-item, self-report scale of self-perceived stress-related behaviour change. Respondents indicate for each stress-sensitive behaviour whether, in the last week, it has been ‘better than normal’ (score 0), ‘normal’ (score 1), or ‘worse than normal’ (score 2). The scale has been validated with the HADS and used to evaluate the immediate emotional and behavioural effects of attending XRM within the UK National Breast Screening Programme. In this context, it was shown to be sensitive to the effects of attending screening (Walker *et al*, 1994) and also having a false-positive recall (Gilbert *et al*, 1998).

*The Spielberger State Anxiety Inventory (SSAI-B*, *brief form)* (Marteau and Bekker, 1992): The SSAI-B is a shortened version (6-item) of the 20-item original scale, which asks how a person feels now and reflects situational factors that may influence anxiety levels (Spielberger *et al*, 1983). The brief form has been validated against the full form and has been shown to have acceptable reliability and validity (Marteau and Bekker, 1992). As in the original scale, each item is scored on a 1–4 scale. If desired, scores on the brief scale can be pro-rated (multiply by 3.333) to render them comparable with scores obtained using the full scale. Norms for the full scale for various populations have been published (Spielberger *et al*, 1983).

*Impact of Events (IoE) scale* (Horowitz *et al*, 1979): This is a 15-item scale, whereby respondents rate the frequency with which they have experienced intrusive images and avoidance behaviour related to a specific life event in the last 7 days. In addition to measuring intrusion and avoidance, total scores can be used to assess total subjective distress. Each item is scored according to the frequency with which the respondent has had the experience described in the previous 7 days (‘not at all’ scores 0, ‘rarely’ scores 1, ‘sometimes’ scores 3, and ‘often’ scores 5). Thus, the total score can range from 0 to 75.

The IoE was administered twice at follow-up, once anchored to XRM, and once anchored to MRI.

Assessments were carried out at the following time points:

*Baseline (4 weeks before the scans)*: Information was collected for sociodemographic characteristics and previous screening experiences, and the HADS and HQ were administered. Women were sent the baseline booklet by post and were asked to return it in advance of screening in the enclosed stamped envelope addressed to the Research Assistant at the Institute of Rehabilitation, University of Hull, UK.

*Immediately before MRI and immediately before XRM*: The HADS, HQ, and SSAI-B were administered. Women were given the pre- and post-scan booklets by radiographers according to a standardised protocol.

*Immediately after MRI and immediately after XRM*: Information was collected regarding acceptability, distress caused by procedure, and satisfaction with procedure, and the SSAI-B was administered. Women were given the pre- and post-scan booklets by radiographers according to a standardised protocol.

*Follow-up:* The HADS, HQ, and IoE were administered and intention to return for screening was assessed. Women were sent the follow-up booklet by post, and they were asked to return it in an enclosed stamped envelope addressed to the Research Assistant at the Institute of Rehabilitation, University of Hull, UK. Women who had not responded within a week were sent reminders and given a further week to respond. Questionnaires received after this time were excluded from the analyses.

### Analysis plan

There were two primary end points, both in year 1: immediate effects of MRI and XRM (as soon as the procedure had been completed), and the delayed effects of MRI and XRM (at 6-week follow-up).

The primary immediate outcome measure was scores on the SSAI-B, and the primary follow-up outcome measurement was total scores on the IoE scale.

Secondary immediate outcome measures of the two procedures were the HADS, HQ, and *ad hoc* items relating to acceptability, distress, and satisfaction. Secondary delayed outcome measures were the HADS, HQ, and *ad hoc* items relating to intention to return.

The MARIBS Study was designed to have adequate power to test the primary study hypothesis, namely, that MRI can be used with equal or better sensitivity than XRM, with an acceptable false-positive rate, rather than to detect differences in psychological variables (Brown *et al*, 2000a, 2000b). However, in terms of primary psychological outcomes, the power to detect a one-point difference between the two procedures using the SSAI-B was 97%, and the power to detect a 1.5-point difference using the IoE total score was also 97%.

Data were analysed using *t*-tests, Mann–Whitney *U*-tests, and *χ*^2^-test as appropriate. α-Value was set at 0.05 for two-tailed tests.

## Results

### Recruitment and retention

**Figure 1:**
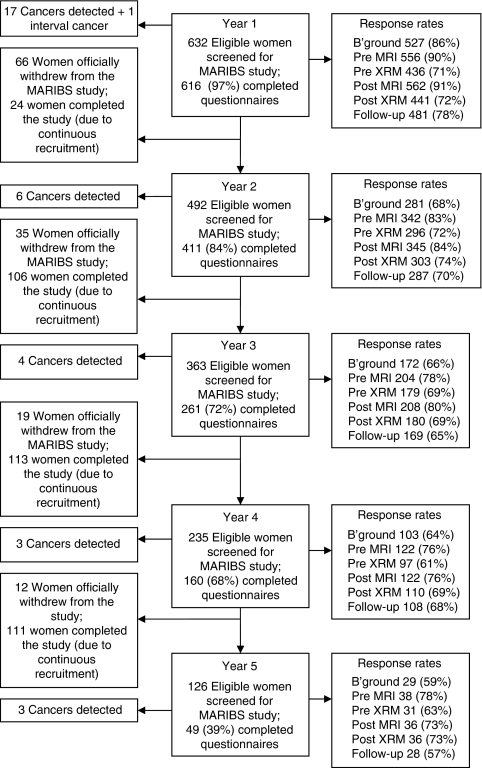
Trial profile. Numbers of women who were screened each year for MARIBS, number of participants in the psychology study, the response rates for each of the assessments (includes the number of women who withdrew or were withdrawn because of a cancer diagnosis), and the number of women who completed the study by year (because of continuous recruitment over the 5-year period).

Over the 5 years of continuing recruitment to the psychology study, 616 women (97% of all women screened for MARIBS) were recruited for their first screening round; 411 (84% of all women screened) returned for year 2 MRI and XRM; 261 (72% of all women screened) in year 3; 160 (68% of all women screened) in year 4, and 49 (39% of all women screened) in year 5. Figure 1 shows the number of women recruited to the psychological arm of the study and the response rates for each of the assessments. It also shows the number of cancers detected each year.

Of all women invited to take part in the MARIBS 58% accepted the invitation following genetic counselling. This acceptance rate has been published previously (Evans *et al*, 2001).

Table 1 describes the sociodemographic details of the study participants, their previous screening experiences, and scores on the psychological measures at baseline (1 month before MRI and XRM).

### Baseline characteristics

Hospital Anxiety and Depression Scale depression scores were highest at baseline, and significantly higher than immediately before MRI (*t*=3.38, *P*=0.001, CI: 0.28–1.05). This difference in depression scores accounts for the significantly higher level of total distress (HADS) at baseline, compared with immediately before MRI (*t*=2.60, *P*=0.009, CI: 0.26–1.87). This difference was not related to having had a previous MRI scan (*t*=−0.74, *P*=0.46, CI: −0.90 to 0.41) or to having previously found MRI distressing (*t*=1.57, *P*=0.12, CI: −0.36 to 3.11). Hospital Anxiety and Depression Scale anxiety scores were relatively constant throughout.

In terms of clinically significant categories on the HADS, there were no significant differences in the proportion of women falling into the normal, borderline, or clinically significant categories of anxiety at baseline compared with pre-MRI values (*χ*^2^=5.27, *P*=0.26, CI: 0.26–0.27), or in the numbers of women falling into the normal, borderline, or clinically significant categories of depression at baseline compared with pre MRI (*χ*^2^=1.11, *P*=0.89, CI: 0.92–0.93).

### Outcomes immediately before and after screening

Table 2 shows the psychological outcomes at screening. In year 1, there were no significant differences in anxiety, depression, or total scores (HADS), or in stress-related behaviour change (HQ) immediately before MRI and immediately before XRM. The primary outcome, anticipatory anxiety (SSAI-B) in year 1, however, was significantly higher before MRI than before XRM (*t*=3.00, *P*=0.003, CI: 0.27–1.30). State anxiety dropped after screening for both MRI (*t*=5.83, *P*<0.0005, CI: 0.92–1.86) and for XRM (*t*=3.18, *P*=0.002, CI: 0.34–1.42), and there was no significant difference in state anxiety after MRI compared with after XRM (*t*=1.075, *P*=0.28, CI: −0.22 to 0.77). The difference in state anxiety before MRI and before XRM was not statistically significant in year 2 (*t*=1.86, *P*=0.063, CI: −0.03 to 1.23), but re-emerged in year 3 (*t*=2.30, *P*=0.022, CI: 0.134–1.75). The difference was not significant for women returning for scans in year 4 (*t*=1.17, *P*=0.242, CI: −0.42 to 1.66).

In year 1, immediately after MRI and XRM, 13.5% of women reported finding MRI ‘extremely’, ‘very’, or ‘moderately’ distressing, compared with 7.8% of women finding XRM ‘extremely’, ‘very’, or ‘moderately’ distressing. This difference was significant (*Z*=−2.83, *P*=0.005). Magnetic resonance imaging scans are rated as less distressing in subsequent years, and there is no statistically significant difference between how distressing women find MRI and XRM in years 2, 3, and 4. If the key category is taken as ‘extremely distressing’, then immediately after MRI, 4.4% of women rated MRI as extremely distressing, compared with 1.1% of women rating XRM extremely distressing immediately after XRM (*χ*^2^=0.554, *P*=0.55).

There were very high levels of overall satisfaction with both procedures (MRI-96.3% (68.4% were ‘very satisfied’) and XRM-97.7% (65.5% were ‘very satisfied’)).

Immediately post-MRI, the least acceptable aspects of the procedure were reported as ‘finding the MRI table uncomfortable’ (69.7% of participants agreed or strongly agreed with this statement), ‘the noise during the MRI scan’ (69.7%), ‘the injection’ (68.6%), ‘the screening centre for MRI being in a place that was difficult to get to’ (66.6%), ‘being worried about going into the scanner’ (54.6%), and ‘feeling uncomfortable lying still’ (54.6%). Immediately post-XRM, the least acceptable aspects of the procedure were ‘worry about being exposed to radiation’ (73.5%), ‘finding the mammogram uncomfortable’ (73.0%), ‘the screening centre for XRM being in a place that was difficult to get to’ (69.6%), ‘the way I had to stand’ (63.6%), and ‘pain’ (62.0%).

### Follow-up at 6 weeks post mammography

Table 3 shows the psychological outcomes at 6-week follow-up. At 6 weeks after the year 1 scans, women were significantly less anxious (HADS; *t*=3.93, *P*<0.0005, CI: −1.75 to 1.48) and less depressed (HADS; *t*=2.00, *P*=0.045, CI: −0.84 to 0.01) than they had been at baseline. Low levels of anxiety and depression were consistently reported at 6 weeks after the scans in subsequent years. In terms of clinically significant categories, there was no significant difference in the proportion of women falling into the HADS normal, borderline, or clinically significant categories of anxiety at baseline compared with follow-up (*χ*^2^=8.23, *P*=0.083, CI: 0.08–0.09). However, there was a significant difference for depression; significantly more women moved from the borderline category of depression into the normal range at follow-up (*χ*^2^=15.3, *P*=0.04, CI: 0.01–0.01).

With regard to the primary outcome (delayed MRI and XRM-related distress (IoE scale)), significantly more women reported intrusive MRI-related thoughts than intrusive XRM-related thoughts (*t*=2.75, *P*=0.006, CI: 0.12–0.72). This accounted for significantly greater total subjective MRI-related distress (IoE) compared with total subjective XRM-related distress (*t*=2.47, *P*=0.014, CI: 0.15–1.33). This effect was not related to previous screening experience: those having had an MRI scan had similar levels of intrusive MRI-related thoughts than those for whom the procedure was new (*t*=1.16, *P*=0.24). The level of avoidant MRI-related thoughts compared with avoidant XRM-related thoughts (IoE) showed a non-significant trend (*t*=1.88, *P*=0.060, CI: −0.01 to 0.62).

In year 2, women reported significantly more intrusive MRI-related thoughts compared with intrusive XRM-related thoughts (*t*=2.73, *P*=0.007, CI: 0.14–0.84), significantly more avoidant MRI-related thoughts compared with XRM-related thoughts (*t*=2.92, *P*=0.004, CI: 0.19–1.00), and more total subjective MRI-related distress compared with total subjective XRM-related distress (*t*=2.96, *P*=0.003, CI: 0.37–1.82). Differences did not reach statistical significance in years 3 and 4.

Six weeks after the year 1 scans, 88% of participants stated that they intended to return for MRI compared with 96.3% for XRM (*Z*=−4.58, *P*<0.0005). This significant difference in intention to re-attend was maintained in years 2 (*Z*=−3.96, *P*<0.0005) and 3 (*Z*=−2.25, *P*=0.024). Intention to return for MRI is strongly related to intrusive thoughts about MRI at follow-up (as assessed using the IoE scale). The mean score for intrusive thoughts about MRI of those intending the return was 8.90 (s.d. 3.32) compared with 13.2 (s.d. 5.32) of those unwilling or unsure about coming back (*t*=7.98, *P*<0.0005, CI: 3.30–5.45). However, it is well known that behavioural intentions do not always translate into behaviour. Unsurprisingly, women who indicated that they did not intend to return for MRI, or who were unsure about whether to re-attend, were significantly more likely not to return for screening (*χ*^2^=8.81, *P*=0.012). However, 4 of the 12 women who indicated that they did not intend to return, and 10 of the 38 women who said they were unsure about returning in year 1, did in fact return for screening in year 2.

Over the course of the study, 27 women declined MRI outright, and 16 women attended MRI, but experienced a ‘technical failure’ (for example, they were too big for the scanner or staff were unable to get venous access for the contrast agent). A total of 106 women withdrew over the course of the study. The most common reasons for withdrawal were receiving a negative gene test; receiving a breast cancer diagnosis; stress/personal reasons; claustrophobia/scan uncomfortable; having undergone prophylactic mastectomy; and not being able to contact participants in subsequent years.

We had been concerned that the order of the scans, that is, whether women received XRM or MRI first, might affect the results. Specifically, the concern was that women would be more anxious for their first scan, irrespective of whether it was XRM or MRI that was carried out first. Unfortunately, it was not practical to randomise individual women to receiving MRI or XRM first. However, some centres undertook to perform MRI first and others XRM first, so that a sub-analyses of order effects could be undertaken. Order effects were not significant. Differences in anxiety and acceptability between screening centres were greater than any effects of receiving MRI or XRM first. The centre with the lowest mean anticipatory anxiety before MRI (as assessed using the SSAI-B) scored 10.7 (s.d. 3.4), compared with the centre with the highest mean pre-MRI anticipatory anxiety score of 16.7 (s.d. 4.8). The difference between these two centres was highly significant (*t*=4.0, *P*<0.0005, CI: 2.98–9.02). The differences in anticipatory anxiety are unrelated to the size of the screening centre. Environmental and interpersonal factors such as the ethos of the centre and communication skills of staff are likely to be more important.

## Discussion

Throughout the study, low levels of psychological morbidity, as assessed by the HADS, were observed. Even at baseline, in which the mean scores for anxiety and depression were highest, only 19.6% of women met the criteria for clinically significant anxiety and 4% met the criteria for clinically significant depression. These figures are broadly representative of HADS scores seen for women in the general UK population (Crawford *et al*, 2001). The significant difference noted in mean scores of HADS depression represents small changes in mean scores with a relatively large sample size. It does not represent more clinically meaningful changes in the numbers of women falling into normal, borderline, or clinically significant categories of distress.

Hospital Anxiety and Depression Scale distress was highest at baseline (1-month before the scans) and lowest at follow-up (6 weeks after the scans). In this study, when they completed the baseline questionnaires, women knew they would be attending for screening in the near future. In the context of the National Breast Screening Programme, we used the HADS to assess mental state before the women were invited to attend screening and found that there was a significant decline when they attended for screening (Walker *et al*, 1994). It may be that in a screening context, HADS scores tend to reduce between the first and second administration. However, the observed findings in this study are consistent with the hypothesis that women may be most psychologically vulnerable at the point of recruitment into the study, and in the lead up to the scans; this has obvious implications for the timing of appropriate information and support. The HADS data are also consistent with the view that the long-term impact of screening on mental health is positive.

Women found MRI to be significantly more distressing than conventional XRM (*ad hoc* rating scale). They were more anxious (STAI-B) before MRI than before XRM. Anxiety dropped significantly during both procedures, and there was no significant difference in state anxiety afterwards. It might have been expected that anticipatory anxiety would habituate with repeated exposure. However, although the difference in anticipatory anxiety was not significant in year 2, it re-emerged in year 3.

In all, 13.5% of women reported that they had found MRI ‘extremely’, ‘very’, or ‘moderately’ distressing compared with only 7.8% of women finding XRM ‘extremely’, ‘very’, or ‘moderately’ distressing. The first MRI scan was the most distressing: the number of women reporting distress fell in subsequent years. It should be noted, however, that both procedures were highly acceptable and that the vast majority of women reporting little or no MRI- or XRM-related distress in year 1 (86.5 and 92.2%, respectively). Moreover, there were very high levels of satisfaction with both procedures (96.3 and 97.7%, respectively).

With regard to the long-term effects of screening, HADS distress was consistently lowest at follow-up (6 weeks after the scans), which suggests that the overall psychological impact of screening was positive. However, with regard to MRI- and XRM-specific distress measured by the IOE scale at follow-up, women reported significantly more intrusive thoughts about MRI and more total MRI-related subjective distress compared with XRM. This persisting MRI-specific distress was not restricted to year 1: 6 weeks after the year 2 scans, women also reported significantly more MRI- than XRM-specific distress.

Six weeks after the scans, the vast majority of women indicated that they planned to return for scans the following year. However, significantly more women expressed reluctance to re-attend for MRI than XRM. This effect was not restricted to the first year, but was present in year 2. In the total cohort, 106 of 838 (12.6%) women were excluded or withdrew before year 1 scans because of logistical problems. The most commonly cited reasons for withdrawing from the study were receiving a negative gene test; receiving a breast cancer diagnosis; stress/personal reasons; claustrophobia/scan uncomfortable; having undergone prophylactic mastectomy; not being able to contact participants in subsequent years.

We believe these results can be generalised to the wider population of women at genetic risk for breast cancer. In the wider MARIBS study, 732 women were screened in year 1 (Warren *et al*, 2005). The psychological arm of the study included 616 of these, and the majority of those 116 who were not included were women who were recruited in the first year of MARIBS, before the psychological study had commenced. On the whole, response rates were good (71–91% in year 1). The bulk of missing data, including most of the women lost to follow-up, came from 1 of the 22 screening centres, where completing questionnaires was difficult due to practical constraints and the screening environment; hence, the data may be even more representative than the trial profile (Figure 1) would suggest.

The categories of women eligible for screening under the UK NICE guidelines (McIntosh *et al*, 2006) reflect closely the inclusion criteria for the MARIBS study. The results are also relevant to higher risk groups in terms of the American Cancer Society guidelines (Saslow *et al*, 2007), although we are unable to comment on possible cross-cultural psychosocial differences.

We had intended to look at the impact of ‘false-positive’ MRI scans, that is, to look at the levels of anxiety generated by being recalled for a second MRI scan, which ultimately turned out to be normal. A second MRI scan was undertaken when an anomalous result was seen on MRI, but not on XRM. There were fewer false-positive MRI scans that had been anticipated because of the better specificity of MRI than had been predicted at the outset of the study. A total of 10.7% of women were recalled for further assessment following MRI (Leach *et al*, 2005), resulting in a total of 85 recalls, 25 of which were positive, in year 1. Psychological data were collected for 27 women recalled for second scans in year 1 and for 11 women who were recalled in year 2. The data did not indicate a significant increase in anxiety before the second scan, although the sample sizes are too small to make any general conclusions about the impact of false-positive MRI scans. However, MRI and/or cancer-related distress were not commonly cited reasons for withdrawing from MARIBS. The reasons for withdrawing were diverse. Overall, in the course of the study, the five most common reasons for withdrawal were negative predictive gene test (*n*=30), the development of breast cancer (*n*=35), personal reasons or stress (*n*=19), claustrophobia (*n*=12), and prophylactic mastectomy (*n*=28).

For logistical and ethical reasons, women who went on for further investigations, such as biopsy, were excluded from the psychology study.

In conclusion, from a psychological point of view, it appears that both MRI and XRM are acceptable techniques for screening the majority of women with a family history of breast cancer, at least within the context of a multicentre randomised controlled trial. Although small changes in mean HADS anxiety scores were noted, there were no clinically meaningful changes in either anxiety or depression throughout the course of screening, as evidenced by changes in the proportion of women falling into normal, borderline, or clinically significant categories. The overwhelming majority of women reported little or no distress and feelings of being satisfied with both procedures. However, in terms of our primary outcomes, we did find that MRI was more distressing than XRM, and women experienced significantly more anticipatory anxiety before MRI and significantly more MRI-related distress 6 weeks later. Most women stated that they intended to re-attend for screening, although significantly more women expressed a reluctance to re-attend for MRI compared with XRM. These effects are most marked for the first MRI, but remain in subsequent years.

Given the proven benefits of MRI screening in this population of high-risk women, these data point to an urgent need to develop and evaluate strategies of providing information and support to women undergoing MRI. These strategies should have the aim of minimising distress and optimising re-attendance, and be realistic within the context of a national screening programme.

## Figures and Tables

**Table 1:** Description of participants (from 527 background questionnaires)

*Sociodemographic characteristics*					
Age (years; *n*=525)	Median, 40	Range, 31–55			
Marital status (*n*=499)	Married/living with a partner, 79.0%	Single, 7.8%	Divorced/separated, 12.4%	Widowed, 0.8%	
Ethnic origin (*n*=525)	White, 97.7%	Pakistani, 0.2%	Indian, 1.3%	Chinese, 0.2%	Other, 0.6%
Age on leaving full-time education (*n*=526)	14–16, 39.5%	16–18, 37.6%	18–21, 10.6%	21+, 12.2%	
Currently employed (*n*=524)	Yes, 76.3%	No, 23.7%			
Have children (*n*=523)	Yes, 85.5%	No, 14.5%			
No of children (*n*=457)	Median, 2	Range, 1–7			
Taking medication					
Anxiety (*n*=495)	Yes, 4.6%	No, 95.4%			
Depression (*n*=505)	Yes, 4.8%	No, 95.2%			
Sleeplessness (*n*=505)	Yes, 3.6%	No, 96.4%			
					
*Screening experiences*					
Previously had MRI scan (*n*=519)	Yes, 25.0%	No, 75.0%			
Found previous MRI scan distressing (*n*=128)	Extremely, 3.9%	Very, 2.3%	Moderately, 6.3%	A little, 14.8%	Not at all, 72.7%
Previously had XRM (*n*=522)	Yes, 82.6%	No, 17.4%			
Found previous XRM scan distressing (*n*=426)	Extremely, 2.3%	Very, 0.2%	Moderately, 5.6%	A little, 18.3%	Not at all, 73.5%
Undertake breast self-examination (BSE; *n*=522)	Yes, 87.2%	No, 12.8%			
Finds BSE distressing (*n*=448)	Extremely, 0.9%	Very, 0.4%	Moderately, 4.7%	A little, 10.9%	Not at all, 83.0%
Feels confident about BSE (*n*=456)	Extremely, 3.3%	Very, 13.2%	Moderately, 51.3%	A little, 20.2%	Not at all, 12.1%
Has clinical breast examinations (CBE; *n*=524)	Yes, 90.6%	No, 9.4%			
Finds CBE distressing (*n*=456)	Extremely, 0.9%	Very, 1.3%	Moderately, 3.9%	A little, 13.2%	No at all, 80.7%
Feels confident about doctors’ CBE ability (*n*=475)	Extremely, 30.7%	Very, 48.2%	Moderately, 18.1%	A little, 1.7%	Not at all, 1.3%
					
*Baseline psychological measures*					
Hospital Anxiety and Depression Scale					
Anxiety (*n*=526)	Mean (s.d.), 6.90 (4.16)	Normal range, 61.8%	Borderline, 18.6%	Clinically significant, 19.6%	
Depression (*n*=526)	Mean (s.d.), 3.49 (3.31)	Normal range, 86.3%	Borderline, 9.7%	Clinically significant, 4.0%	
Total distress (*n*=526)	Mean (s.d.), 10.4 (6.79)				
Health questionnaire (*n*=527)	Mean (s.d.), 7.17 (1.83)				

Abbreviations: MRI=magnetic resonance imaging; XRM=X-ray mammography.

**Table 2:** Psychological outcomes at screening (values are given as mean (s.d.))

	**Pre MRI**	**Post MRI**	**Pre XRM**	**Post XRM**
**Year 1**	**556 Questionnaires completed**	**562 Questionnaires completed**	**436 Questionnaires completed**	**441 Questionnaires completed**
*Primary outcome*				
SSAI-B	**12.1 (4.01)**; *n*=546, *t*=3.00, *P*=0.003, CI 0.27–1.30	**10.7 (3.84)**; *n*=542	**11.3 (4.09)**; *n*=429	**10.5 (3.94)**; *n*=426
				
*Secondary outcomes*				
HADS anxiety	6.50 (4.17) Normal, 62.5% Borderline, 20.2% Clinically significant, 17.3%* n*=555		6.49 (4.14) Normal, 62.4% Borderline, 17.9% Clinically significant, 19.7%* n*=431	
HADS depression	2.83 (3.14) Normal, 89.7% Borderline, 8.2% Clinically significant, 2.2%* n*=551		2.90 (3.21) Normal, 89.6% Borderline, 7.0% Clinically significant, 3.5%* n*=431	
HADS total distress	9.32 (6.68); *n*=551		9.37 (6.66); *n*=431	
HQ	7.11 (1.87); *n*=552		6.93 (1.69); *n*=428	
				
**Year 2**	**342 Questionnaires completed**	**345 Questionnaires completed**	**296 Questionnaires completed**	**303 Questionnaires completed**
SSAI-B	11.4 (3.94); *n*=335	10.3 (3.76); *n*=329	10.8 (4.08); *n*=281	10.3 (3.84); *n*=296
HADS anxiety	5.92 (4.29) Normal, 67.6% Borderline, 16.8% Clinically significant, 15.6%* n*=340		5.71 (4.18) Normal, 71.4% Borderline, 13.9% Clinically significant, 14.6%* n*=294	
HADS depression	2.69 (3.43) Normal, 89.0% Borderline, 7.4% Clinically significant, 3.6%* n*=337		2.46 (3.23) Normal, 89.5% Borderline, 7.8% Clinically significant, 2.7%* n*=294	
HADS total distress	8.61 (6.99); *n*=337		8.15 (6.71); *n*=294	
HQ	6.98 (1.96); *n*=336		7.00 (1.87); *n*=292	
				
**Year 3**	**204 Questionnaires completed**	**208 Questionnaires completed**	**179 Questionnaires completed**	**180 Questionnaires completed**
SSAI-B	**11.4 (4.05)**; *n*=198, *t*=2.30, *P*=0.022, CI: 0.22–0.77	**10.0 (3.84)**; *n*=205	**10.5 (3.86)**; *n*=178	**9.99 (3.61)**; *n*=178
HADS anxiety	5.62 (4.19) Normal, 70.6% Borderline, 15.9% Clinically significant, 13.4%* n*=201		5.49 (4.00) Normal, 72.7% Borderline, 14.8% Clinically significant, 12.5%* n*=176	
HADS depression	2.58 (3.30) Normal, 87.5% Borderline, 9.5% Clinically significant, 3.0%* n*=200		2.58 (3.24) Normal, 87.6% Borderline, 10.7% Clinically significant, 1.7%* n*=177	
HADS total distress	8.20 (6.82); *n*=198		8.09 (6.56); *n*=174	
HQ	6.90 (1.74); *n*=203		6.89 (1.79); *n*=178	
				
**Year 4**	**122 Questionnaires completed**	**122 Questionnaires completed**	**97 Questionnaires completed**	**110 Questionnaires completed**
SSAI-B	10.8 (3.81); *n*=119	9.59 (3.18); *n*=116	10.1 (3.92); *n*=92	9.72 (3.11); *n*=109
HADS anxiety	5.20 (4.05) Normal, 71.4% Borderline, 15.1% Clinically significant, 13.4%* n*=119		4.98 (3.91) Normal, 74.2% Borderline, 15.5% Clinically significant, 10.3%* n*=97	
HADS depression	2.53 (3.17) Normal, 89.3% Borderline, 8.3% Clinically significant, 2.5%* n*=121		2.40 (3.15) Normal, 88.4% Borderline, 8.4% Clinically significant, 3.2%* n*=96	
HADS total distress	7.80 (6.66); *n*=118		7.33 (6.42); *n*=95	
HQ	6.88 (1.73); *n*=121		6.96 (1.82); *n*=96	

Abbreviations: HADS=Hospital Anxiety and Depression Scale; HQ=Health Questionnaire; MRI=magnetic resonance imaging; SSAI-B=Spielberger State Anxiety Inventory; XRM=X-ray mammography. Bolded figures denote significant results.

**Table 3:** Psychological outcomes at 6-week follow-up

	**Year 1**	**Year 2**	**Year 3**	**Year 4**
	**481 Questionnaires completed**	**287 Questionnaires completed**	**169 Questionnaires completed**	**108 Questionnaires completed**
*Primary outcomes*				
IoE total subjective distress MRI	**19.3 (7.01)**; *n*=443, *t*=2.47, *P*=0.014, CI: 0.15–1.33^a^	**18.8 (6.94)**; *n*=270, *t*=2.96, *P*=0.003, CI: 0.37–1.82^a^	17.8 (5.37); *n*=165	17.8 (5.82); *n*=104
IoE intrusive thoughts MRI	**9.15 (3.55)**; *n*=446, *t*=2.75, *P*=0.006, CI: 0.12–0.72^a^	**8.73 (3.32)**; *n*=272, *t*=2.73, *P*=0.007, CI: 0.14–0.84^a^	8.28 (2.60); *n*=167	8.31 (2.73); *n*=108
IoE avoidant thoughts MRI	10.2 (3.84); *n*=444	**10.1 (3.93)**; *n*=271, *t*=2.92, *P*=0.004, CI 0.19–1.00)^a^	9.55 (3.08); *n*=165	9.54 (3.43); *n*=104
IoE total subjective distress XRM	18.6 (5.24); *n*=416	17.8 (5.25); *n*=255	17.4 (4.46); *n*=154	17.2 (4.43); *n*=96
IoE intrusive thoughts XRM	8.72 (2.66); *n*=425	8.26 (2.48); *n*=258	7.94 (2.03); *n*=156	7.83 (1.87); *n*=98
IoE avoidant thoughts XRM	9.88 (2.93); *n*=419	9.59 (3.09); *n*=257	9.47 (2.84); *n*=154	9.43 (2.99); *n*=96
				
*Secondary outcomes*				
HADS anxiety	**5.87 (4.09)** Normal, 67.7% Borderline, 19.0% Clinically significant, 13.3%* n*=474, *t*=3.93, *P*<0.0005, CI: −1.75 to 1.48^a^ (compared with baseline)	5.50 (3.99) Normal, 70.4% Borderline, 17.0% Clinically significant, 12.6%* n*=277	5.35 (3.85) Normal, 71.2% Borderline, 19.0% Clinically significant, 9.8%* n*=163	5.11 (4.22) Normal, 73.8% Borderline, 14.0% Clinically significant, 12.1%* n*=107
HADS depression	**3.07 (3.38)** Normal, 87.1% Borderline, 8.9% Clinically significant, 4.0%* n*=473, *t*=2.00, *P*<0.045, CI: −0.84 to 0.01^a^ (compared with baseline)	2.92 (3.49) Normal, 88.1% Borderline, 8.8% Clinically significant, 3.1%* n*=261	2.61 (3.12) Normal, 87.3% Borderline, 10.2% Clinically significant, 2.4%* n*=166	2.80 (3.71) Normal, 88.8% Borderline, 7.5% Clinically significant, 3.7%* n*=107
HADS total distress	**8.93 (6.87)**; *n*=470, *t*=3.30, *P*=0.001 CI: 0.59–2.33^a^ (compared with baseline)	8.69 (6.82) *n*=256	7.96 (6.39); *n*=166	7.83 (7.19); *n*=107
HQ	6.96 (2.07); *n*=477	6.86 (2.29); *n*=264	6.80 (1.72); *n*=166	6.74 (2.00); *n*=108
Intention to return for MRI	Yes, **88.0%** No, **2.6%** Unsure, **8.4%*** n*=454, *Z*=−4.58, *P*<0.0005^b^	Yes, **89.1%** No, **5.8%** Unsure, **5.1%*** n*=274, *Z*=−3.96, *P*<0.0005^b^	Yes, **94.6%** No, **2.4%** Unsure, **3.0%*** n*=166, *Z*=−2.25, *P*=0.024^b^	Yes, 89.6% No, 6.6% Unsure, 3.8%* n*=106
Intention to return for XRM	Yes, **96.3%** No, **2.1%** Unsure, **1.6%*** n*=434	Yes, **95.5%** No, **1.9%** Unsure, **2.6%*** n*=267	Yes, **98.1%** No, **0.6%** Unsure, **1.3%*** n*=156	Yes, 92.7% No, 5.2% Unsure, 3.1%* n*=96

Abbreviations: HADS=Hospital Anxiety and Depression Scale; HQ=Health Questionnaire; IoE=Impact of Events Scale; MRI=magnetic resonance imaging; XRM=X-ray mammography.

Values given in bold denote significant results.

a *t*-Test used to compare means.

b Mann–Whitney *U*-test used to compare percentages.

## References

[bib1] Anderson J, Walker LG (2002) Psychological aspects of MRI breast screening in women at high risk of breast cancer. In Breast MRI in Practice, Warren R, Coulthard A (eds). Martin Dunitz: London

[bib2] Antoniou A, Pharoah PD, Narod S, Risch HA, Eyfjord JE, Hopper JL, Loman N, Olsson H, Johannsson O, Borg A, Pasini B, Radice P, Manoukian S, Eccles DM, Tang N, Olah E, Anton-Culver H, Warner E, Lubinski J, Gronwald J, Gorski B, Talinius H, Thorlacius S, Eerola H, Nevanlinna H, Syrjakoski K, Kallioniemi OP, Thompson D, Evans C, Peto J, Lalloo F, Evans DG, Easton DF (2003) Average risks of breast and ovarian cancer associated with BRCA1 or BRCA2 mutations detected in case series unselected for family history. Am J Hum Gen 72: 1117–1130 10.1086/375033 PMC1180265 12677558

[bib20] Brown J, Coulthard A, Dixon AK, Dixon JM, Easton DF, Eeles RA, Evans DG, Gilbert FG, Hayes C, Jenkins JP, Leach MO, Moss SM, Padhani AP, Pointon LJ, Ponder BA, Sloane JP, Turnbull LW, Walker LG, Warren RM, Watson W (2000a) Rationale for a national multi-centre study of magnetic resonance imaging (MRI) screening in women at genetic risk of breast cancer. Breast 9(2): 72–77 14731702 10.1054/brst.2000.0135

[bib21] Brown J, Coulthard A, Dixon AK, Dixon JM, Easton DF, Eeles RA, Evans DG, Gilbert FG, Hayes C, Jenkins JP, Leach MO, Moss SM, Padhani AP, Pointon LJ, Ponder BA, Sloane JP, Turnbull LW, Walker LG, Warren RM, Watson W (2000b) Protocol for a national multi-centre study of magnetic resonance imaging (MRI) screening in women at genetic risk of breast cancer. Breast 9(2): 78–82 14731703 10.1054/brst.2000.0136

[bib3] Crawford JR, Henry JD, Crombie C, Taylor EP (2001) Normative data for the HADS from a large non-clinical sample. Br J Clin Psychol 40(4): 429–434 11760618 10.1348/014466501163904

[bib4] Evans DGR, Lalloo F, Shenton A, Boggis C, Howell A (2001) Uptake of screening and prevention trials in women at very high risk of breast cancer. Lancet 358: 889–890 11567707 10.1016/S0140-6736(01)06039-1

[bib5] Gilbert FJ, Cordiner CM, Affleck IR, Hood DB, Mathieson D, Walker LG (1998) Breast screening: the psychological costs of false positive recall in women with and without a history of breast cancer. Eur J Cancer 34: 2010–2014 10070302 10.1016/s0959-8049(98)00294-9

[bib6] Gilbert FJ, Warren RM, Kwan-Lim G, Thompson DJ, Eeles RA, Evans DG, Leach MO, United Kingdom Magnetic Resonance Imaging in Breast Screening (MARIBS) Study Group (2009) Cancers in BRCA1 and BRCA2 carriers and in women at high risk for breast cancer: MR imaging and mammographic features. Radiology 252: 358–368 19703879 10.1148/radiol.2522081032

[bib7] Griebsch I, Brown J, Boggis C, Dixon A, Dixon M, Easton D, Eeles R, Evans DG, Gilbert FJ, Hawnaur J, Kessar P, Lakhani SR, Moss SM, Nerurkar A, Padhani AR, Pointon LJ, Potterton J, Thompson D, Turnbull LW, Walker LG, Warren R, Leach MO, UK Magnetic Resonance Imaging in Breast Screening (MARIBS) Study Group (2006) Cost effectiveness of screening with contrast enhanced magnetic resonance imaging *vs* X-ray mammography of women at high familial risk of breast cancer. Br J Cancer 95: 801–810 17016484 10.1038/sj.bjc.6603356 PMC2360541

[bib8] Hermann C (1997) International experiences with the Hospital Anxiety and Depression Scale – a review of validation cases and clinical results. J Psychosom Res 42: 17–41 9055211 10.1016/s0022-3999(96)00216-4

[bib9] Horowitz M, Wilner N, Alvarez W (1979) Impact of events scale: a measure of subjective stress. Psychosom Med 41(3): 209–218 472086 10.1097/00006842-197905000-00004

[bib10] Katz RC, Wilson L, Frazer N (1994) Anxiety and its determinants in patients undergoing magnetic resonance imaging. J Behav Ther Exp Psychiatry 25: 131–134 7983222 10.1016/0005-7916(94)90005-1

[bib12] Liberman L (2004) Breast cancer screening with MRI – what are the data for patients at high risk? N Engl J Med 351: 497–500 15282358 10.1056/NEJMe048117

[bib13] Lord SJ, Lei W, Craft P, Cawson JN, Morris I, Walleser S, Griffiths A, Parker S, Houssami N (2007) A systematic review of the effectiveness of magnetic resonance imaging (MRI) as an addition to mammography and ultrasound in screening young women at high risk of breast cancer. Eur J Cancer 43: 1905–1917 17681781 10.1016/j.ejca.2007.06.007

[bib14] Leach MO, Brown J, Coulthard A, Dixon AK, Dixon JM, Easton D, Eeles RA, Evans DG, Gilbert FJ, Kessar P, Lakhani SR, Moss S, Nerurkar A, Padhani AR, Potterton AJ, Ponder BAJ, Sloane J, Turnbull LW, Walker LG, Warren RML (2005) Screening with magnetic resonance imaging and mammography of a UK population at high familial risk of breast cancer: a prospective multicentre cohort study (MARIBS). Lancet 365: 1769–1778 15910949 10.1016/S0140-6736(05)66481-1

[bib15] Marteau TM, Bekker H (1992) The development of a six-item short form of the state scale of the Speilberger State-Trait Anxiety Inventory (STAI). Br J Clin Psychol 31(3): 301–306 1393159 10.1111/j.2044-8260.1992.tb00997.x

[bib16] McIntosh A, Shaw C, Evans G, Turnbull N, Bahar N, Barclay M, Easton D, Emery J, Gray J, Halpin J, Hopwood P, McKay J, Sheppard C, Sibbering M, Watson W, Wailoo A, Hutchinson A (2004 updated 2006) Clinical Guidelines and Evidence Review for The Classification and Care of Women at Risk of Familial Breast Cancer. NICE guideline CG041 National Collaborating Centre for Primary Care/University of Sheffield: London

[bib17] McIsaac HK, Thordarson DS, Shafran R, Rachman S, Poole G (1998) Claustrophobia and the magnetic resonance imaging procedure. J Behav Med 21(3): 255–267 9642571 10.1023/a:1018717016680

[bib18] Saslow D, Boetes C, Burke W, Harms S, Leach MO, Lehman CD, Morris E, Pisano E, Schnall M, Sener S, Smith RA, Warner E, Yaffe M, Andrews KS, Russell CA. (2007) American Cancer Society guidelines for breast screening with MRI as an adjunct to mammography. CA Cancer J Clin 57: 75–89 17392385 10.3322/canjclin.57.2.75

[bib19] Spielberger CD, Gorsuch RL, Lushene RD (1983) Manual for the State-Trait Anxiety Inventory – Form Y. Consulting Psychologists Press: Palo Alto, CA

[bib22] van Dooren S, Seynaeve C, Rijnsburger AJ, Duivenvoorden HJ, Essink-Bot ML, Tilanus-Linthorst MM, Klijn JG, de Koning HJ, Tibben A (2005) Exploring the course of psychological distress around two successive control visits in women at hereditary risk for breast cancer. Eur J Cancer 41: 1416–1425 15913982 10.1016/j.ejca.2005.03.020

[bib23] Walker J, Postma K, McHugh GS, Rush R, Coyle B, Strong V, Sharpe M (2007) Performance of the hospital anxiety and depression scale as a screening tool for major depressive disorder in cancer patients. J Psychosom Res 63: 83–91 17586341 10.1016/j.jpsychores.2007.01.009

[bib24] Walker LG, Cordiner CM, Gilbert FJ, Needham G, Deans HE, Affleck IR, Hood DB, Mathieson AK, Ah-See AK, Eremin O (1994) How distressing is attendance for routine breast screening. Psycho-Oncology 3: 299–304

[bib25] Warner E (2004) Intensive radiologic surveillance: a focus on the psychological issues. Ann Oncol 15(Suppl 1): i43–i47 15280187 10.1093/annonc/mdh657

[bib26] Warren R, Hayes C, Pointon L, Hoff R, Gilbert FJ, Padhani AR, Rubin C, Kaplan G, Raza K, Wilkinson L, Hall-Craggs M, Kessar P, Rankin S, Dixon AK, Walsh J, Turnbull L, Britton P, Sinnatamby R, Easton D, Thompson D, Lakhani SR, Leach MO, UK MRC study of MRI screening for breast cancer in women at high risk (MARIBS) (2006a) A test of performance of breast MRI interpretation in a multicentre screening study. Magn Reson Imaging 24(7): 917–929 16916709 10.1016/j.mri.2006.03.004

[bib27] Warren R, Pointon L, Thompson D, Hoff R, Gilbert FJ, Padhani A, Easton D, Lakhani SR, Leach MO, UK Magnetic Resonance Imaging in Breast Screening (MARIBS) Study Group (2005) Reading protocol for dynamic contrast-enhanced MR images of the breast: sensitivity and specificity analysis. Radiology 236(3): 779–788 16118160 10.1148/radiol.2363040735

[bib28] Warren R, Thompson D, Pointon LJ, Hoff R, Gilbert FJ, Padhani AR, Easton DF, Lakhani SR, Leach MO (2006b) Evaluation of a prospective scoring system designed for a multicentre breast MR imaging screening study. Radiology 239(3): 677–685 16714457 10.1148/radiol.2393042007

[bib29] Watson EK, Henderson BJ, Brett J, Bankhead C, Austoker J (2005) The psychological impact of mammographic screening on women with a family history of breast cancer – a systematic review. Psycho-Oncology 14: 939–948 15744777 10.1002/pon.903

[bib30] Zigmond AS, Snaith RP (1983) The hospital anxiety and depression scale. Acta Psychiatr Scand 67: 361–370 6880820 10.1111/j.1600-0447.1983.tb09716.x

